# Estimation of the Relative Sensitivity of qPCR Analysis Using Pooled Samples

**DOI:** 10.1371/journal.pone.0093491

**Published:** 2014-04-10

**Authors:** Ana Muniesa, Chelo Ferreira, Héctor Fuertes, Nabil Halaihel, Ignacio de Blas

**Affiliations:** 1 Department of Animal Pathology, Facultad de Veterinaria, Universidad de Zaragoza, Zaragoza, Spain; 2 Department of Applied Mathematics, Facultad de Veterinaria, Universidad de Zaragoza, Zaragoza, Spain; University of Brighton, United Kingdom

## Abstract

The high sensitivity of qPCR makes it a desirable diagnostic method in epidemiological surveillance programs. However, due to high costs, the use of pooling has been suggested. In this paper, an algorithm based on the Montecarlo method has been designed and implemented. The algorithm had been tested in many different situations, and finally it was validated with a real dataset. Moreover, based on the results obtained and depending on pooling conditions, a drastic decrease of sensitivity is observed.

## Introduction

The Polymerase Chain Reaction (PCR) is one of the most powerful technologies in molecular biology. Using PCR, specific sequences within a DNA or cDNA template can be copied, or “amplified”, many thousand- to a million- fold. PCR is a technique requiring a specific fragment of DNA, and it is useful for different applications: to identify anomalies in the sequence of nucleotides that point to possible genetic diseases[1], to identify an individual or to determine their relationships with others or to detect the presence of DNA of microorganisms useful in the diagnosis of disease or for testing the effectiveness of a treatment [2].

In traditional (endpoint) PCR, detection of the amplified sequence are performed at the end of the reaction after the last PCR cycle, and involve post-PCR analysis such as gel electrophoresis and image analysis.

**Figure 1:**
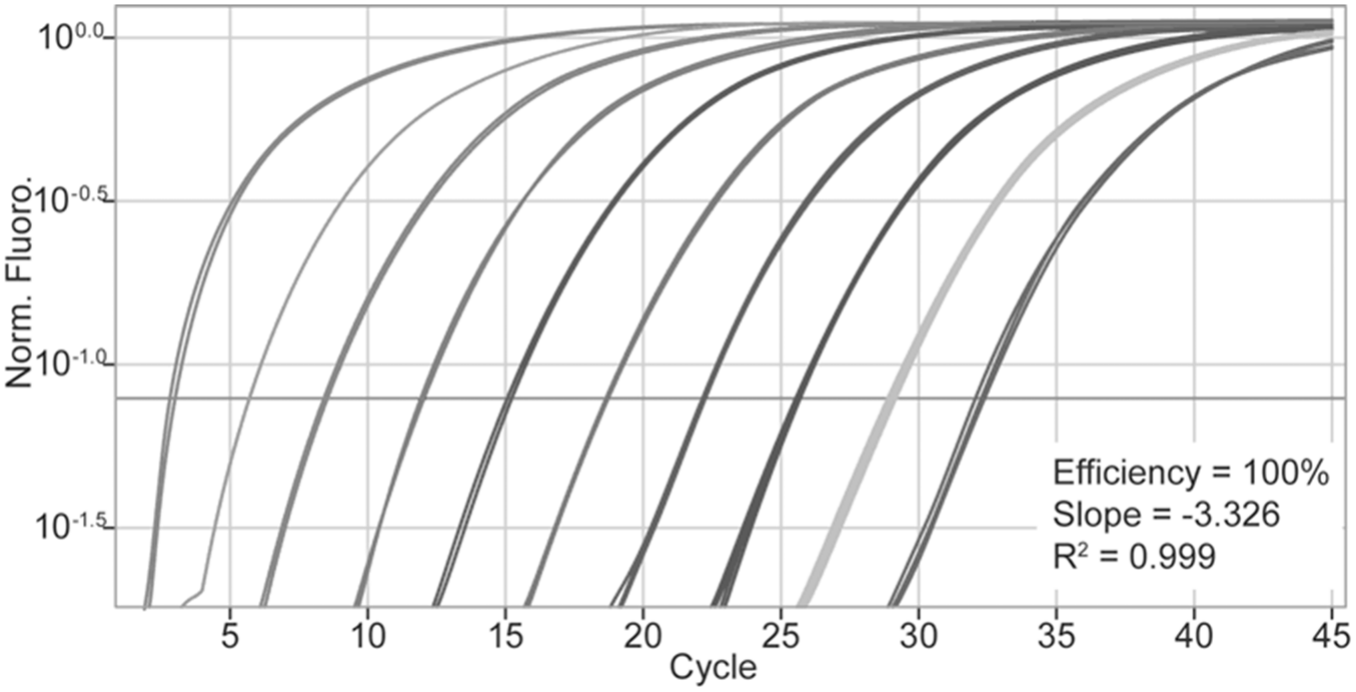
Amplification plots represent the accumulation of product over the duration of the real-time PCR experiment.

In real-time quantitative PCR (qPCR), the amount of PCR product is measured at each cycle by the use of fluorescent markers that are incorporated into the PCR product [3], [2]. The increase in fluorescent signal is directly proportional to the number of PCR product molecules (amplicons) generated in the exponential phase of the reaction. Fluorescent reporters used include double-stranded DNA (dsDNA)-binding dyes, or dye molecules attached to PCR primers or probes that are incorporated into the product during amplification. The change in fluorescence over the course of the reaction is measured by an equipment that combines thermal cycling with scanning capability. By plotting fluorescence against the cycle number, the qPCR equipment generates an amplification plot that represents the accumulation of product over the duration of the entire PCR reaction (Figure 1).

The cycle threshold (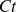) or cycle quantification (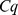) records the cycle when the sample fluorescence exceeds a chosen threshold above background fluorescence. This value is correlated with the number of copies of the target sequence originally present in the reaction mixture [4]. The samples with a high number of initial copies of target nucleic acid, are detected sooner and therefore they will have low 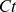 values (usually around 20–25). However, those samples with very low numbers of copies are later detected, and the 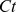 values are above 30–35 [5]. The sample is defined as positive when the 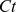 analyzed by the qPCR technique is less than the established 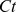 (threshold value). In other cases it is consider as negative.

Using the Standard Curve Method based on known quantities, it is possible to extrapolate a value of a sample. The target DNA gene copies in the pathogen are to be considered to determine the absolute number of the agent in the processed sample, so qPCR provides us the number of copies of a particular pathogen obtained from a sample of an infected individual. The slope of the linear regression curve determines the efficiency of amplification, which is 100% if a dilution of 1∶2 results in a 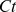 difference of 1 [6].

Currently PCR is the best-known and most successfully implemented diagnostic molecular technology. PCR, specifically qPCR, can detect slow-growing or difficult-to-culture microorganisms and can be used in situations in which clinical microbiology diagnostic procedures are inadequate, time-consuming, difficult, expensive, or hazardous to laboratory staff [2]. The analytical specificity and sensitivity of qPCR assay is considered as perfect for diagnostic of clinical cases (i.e. identification of bovine mastitis pathogens [7]). A general review over the use of qPCR in clinical microbiology testing showed an increased specificity and sensitivity over standard serological tests or culturing methods [8], and for these reasons qPCR is considered as “gold standard” for direct diagnosis in most of pathogens.

The high sensitivity of qPCR makes it as a desirable diagnostic method to use in epidemiological surveillance programs in animal health [9], [10], but qPCR is a relatively expensive technique that limits its generalized application. In order to minimize this problem, the use of pooled samples has been suggested [10]. Thus, it can result in major savings (consumables and labor), and reduced costs [8], [11]. So pooling is now routinely used for health status monitoring purposes.

The theoretical probability of including at least one infected individual in a pool (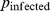) is increased when the pool size (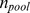) is bigger and the prevalence (P) is higher. This probability is calculated as [12], [13]:
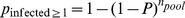
(1)


However, we carefully note the significant decrease in pooled sensitivity due to the dilution effect. Two factors should be taken into account: the low proportion of infected samples in the pool (i.e. pools of big size from a population with low prevalence) and the low number of DNA copies of the infected individuals (i.e. low pathogen loads in individuals from asymptomatic populations [8]). Unfortunately, this is a common scenario of most of the epidemiological surveillance programmes: low prevalence of asymptomatic infected animals in the investigated population.

In most of cases qPCR is considered as gold standard (it means that sensitivity and specifity are perfect). However, it is not completely true and the accuracy may be unknown, so it would be possible to estimate the relative sensitivity of pooled qPCR, assuming individual qPCR as gold standard. An algebraic solution of this problem is not possible and a simulation procedure is suggested.

The objective of this paper is to estimate the relative sensitivity (*rS*) of a qPCR analysis of a pooled sample.

## Materials and Methods

We have designed the following stochastical algorithm using the Montecarlo method. For the later convenience, we define the following variables:

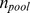
: pool size
- *iCt*: cycle threshold (defined for diagnosis of an individual sample)
- *mCt*: mean of 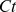 in an infected and asymptomatic population.
- *difCt*  =  *iCt*-*mCt*
- *sCt*: standard deviation of 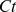 in an infected and asymptomatic population
- *P*: prevalence of infection in an asymptomatic population

Firstly, we define a pooled sample as a mix of *npool* individual samples.

For each of the individual samples (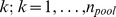), the infection status (infected/not infected) is randomly determinated as a function of the prevalence (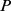).

For non-infected individual samples, the pathogen load is assumed as zero (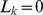). For infected samples we calculate a random 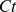 assuming a gaussian distribution with a mean (*mCt*) and standard deviation (*sCt*) given
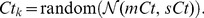
(2)


Next, the load of an infected individual sample (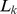) is estimated by 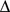
Ct-method [14].
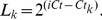
(3)


The load of the pooled sample (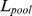) is estimated as the average of the individual samples loads (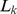)
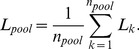
(4)


Then, the 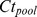 is given as
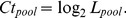
(5)


As we have explained in the introduction section, when the 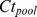 is lower than the 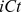 the pool is considered as positive; in other case, as negative.

During the simulation, the algorithm is iterated until a desired quantity of infected pools (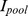) is reached. So, the number of simulations depends on the required precision for the relative sensitivity. We consider as infected pool any pool that included at least one infected individual sample. Moreover, following the previous criteria, the number of positive infected pools (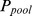) is determinated. Therefore, the relative sensitivity is estimated as [12]

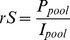
(6)


The algorithm is implemented with *php* language (it is possible to obtain the code just asking for the authors). It has also been implemented in a web page (http://www.winepi.net/f302.php?ID=2) in order to make it available to the scientific community and biomedical practitioners. Accuracy of the results depends on the number of iterations.

In order to validate the algorithm, we have used published data about prevalence of PRRS and QPCR results [8].

## Results and Discussion

The numerical experiments have been given for the all combinations of the next values of the variables 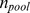 (2, 5, 10, 20), *difCt* (2, 4, 6), 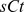 (0.5, 1, 2), and 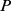 (1, 2, 3, 4, 5, 10, 15,…, 50). The results showed in the graphics are the average of three simulations of 10000 iterations each one.

**Figure 2:**
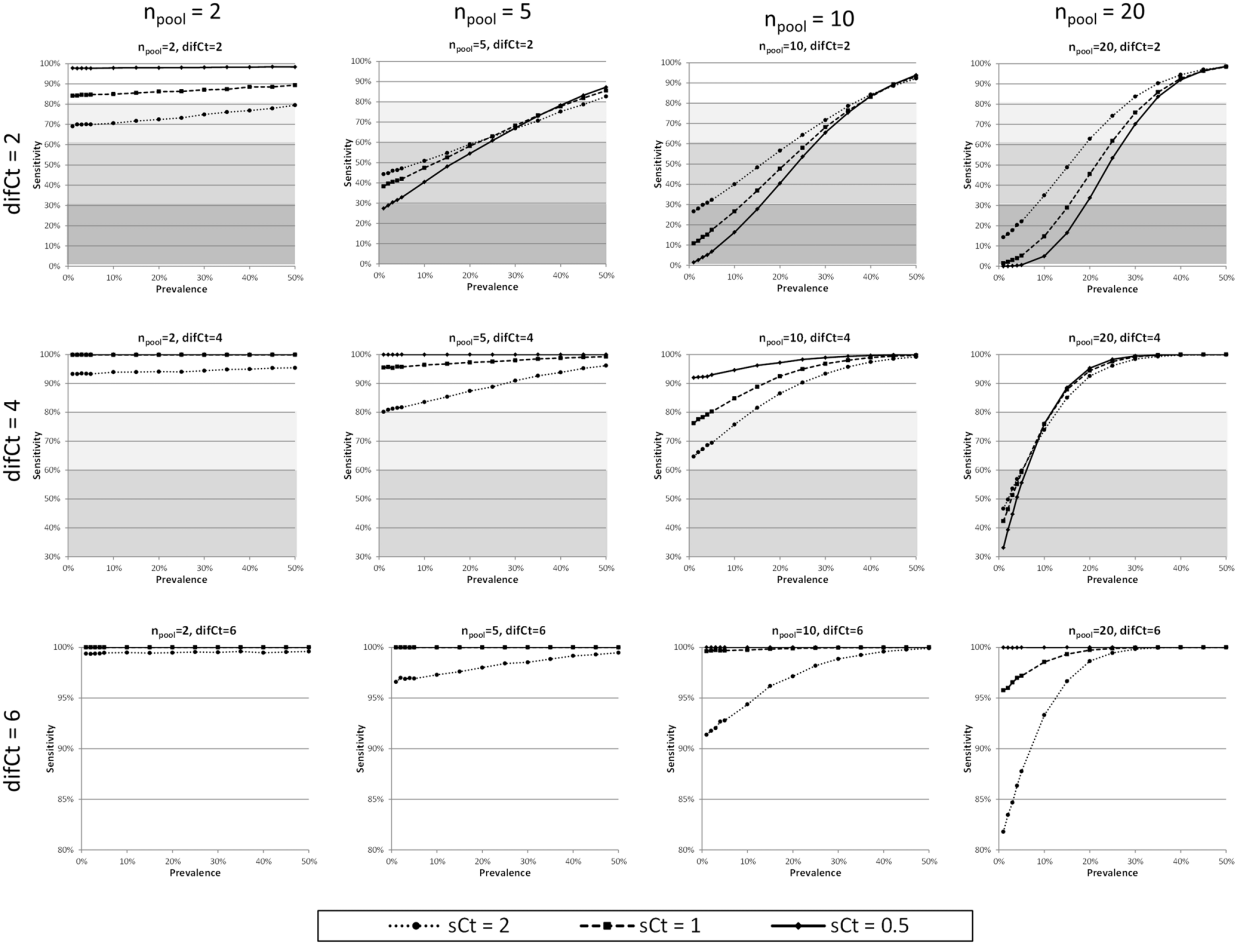
Relative sensitivities corresponding to the simulation results for different scenarios.

By direct observation of the Figure 2, we can extract the following statements:

- Both, low 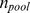 as well as high *difCt*, provide high sensitivity.
- With high values of *difCt* (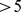), the influence of 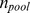 is low, except for high 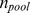 with low prevalence.
- The decreasing values of *difCt*, the increasing influence of 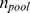. Therefore, higher values of 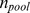, and low prevalence, lower values of the sensitivity.
- The extreme cases are situated at the right upper zone of the Figure 2 (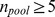 and *difCt
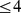
*). The sensitivity values are only acceptable for very high prevalence (highly endemic diseases and epidemic outbreaks).
- Finally, the lower prevalence, the higher effect of the standard deviation effect.

In order to assess the consequences of pooling, we used real data from the experimental work of Gerber et al[8], about the qPCR diagnosis of PRRSv with pooled samples. Based on the individual diagnostic results from serum, the prevalences of infection, for days 1, 3, 5, 7, 14 and 21 post-infection (p.i.), were calculated. And the variabilities of Ct, in all samples globally and in a group defined as low load, were estimated.

**Figure 3:**
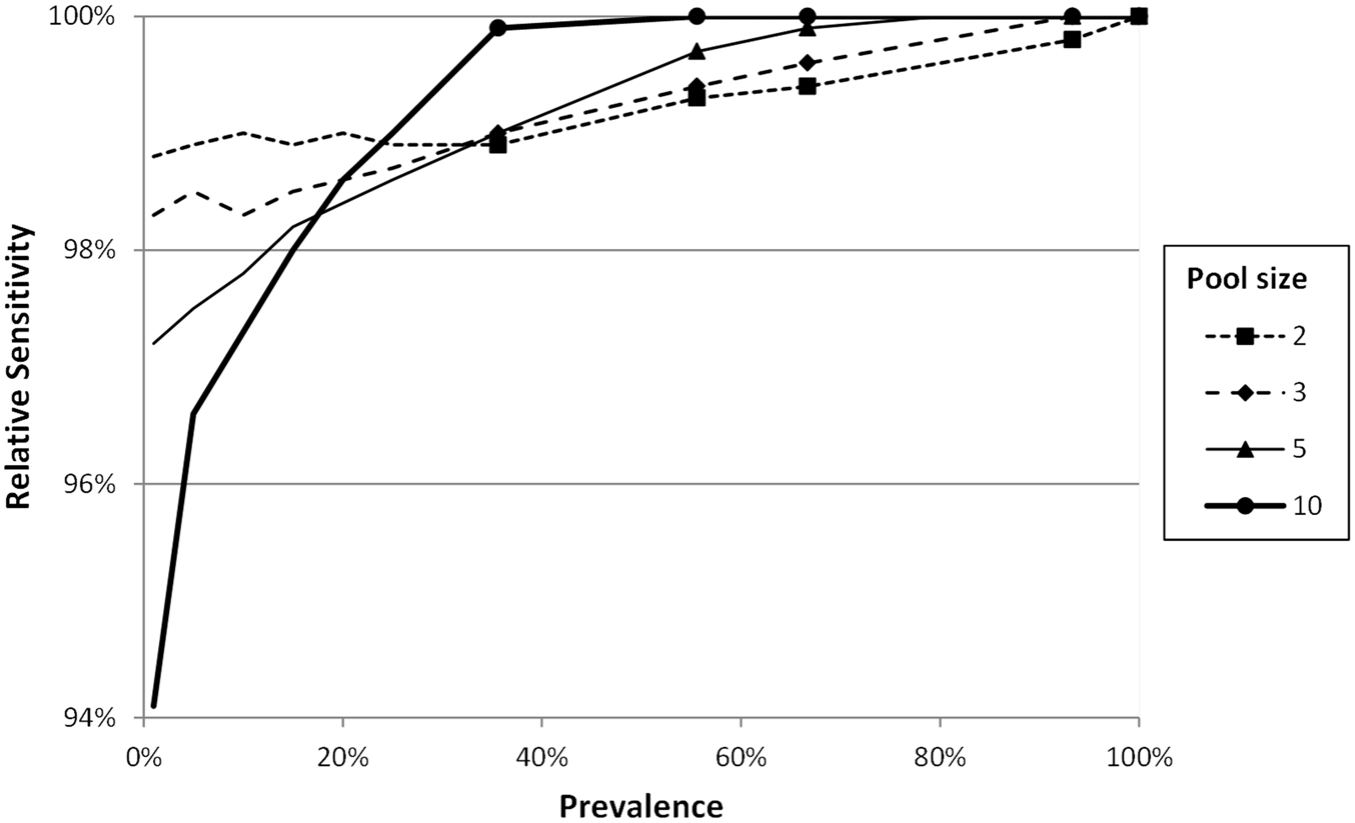
Relative sensitivities corresponding to a pooled diagnostic for PRRSv using global samples of *Ct* (*mCt* = 27.75, *sCt* = 4.625) (data from Gerber et al, 2013)[8].

Firstly, the observed prevalences varied from 66.7% (day 1 p.i.) to 93.3
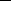
100% during the acute phase (days 3, 5 & 7 p.i.). Then, they decreased progressively to 55.6% (day 14 p.i.) and 35.6% (day 21 p.i.). When we estimated the global *Ct* (*mCt* = 27.75, *sCt* = 4.625), the high variability of the pathogen load is observed. Next, we applied our algorithm, for prevalences from 0% to 100%, to estimate the relative sensitivity in these conditions (Figure 3). In that figure, the specific values corresponding to the prevalences in 1, 3, 5, 7, 14, 21 days p.i, were marked.

The relative sensitivities calculated with our method were over to 98%. Therefore, it is consistent with the results of [8].

**Figure 4:**
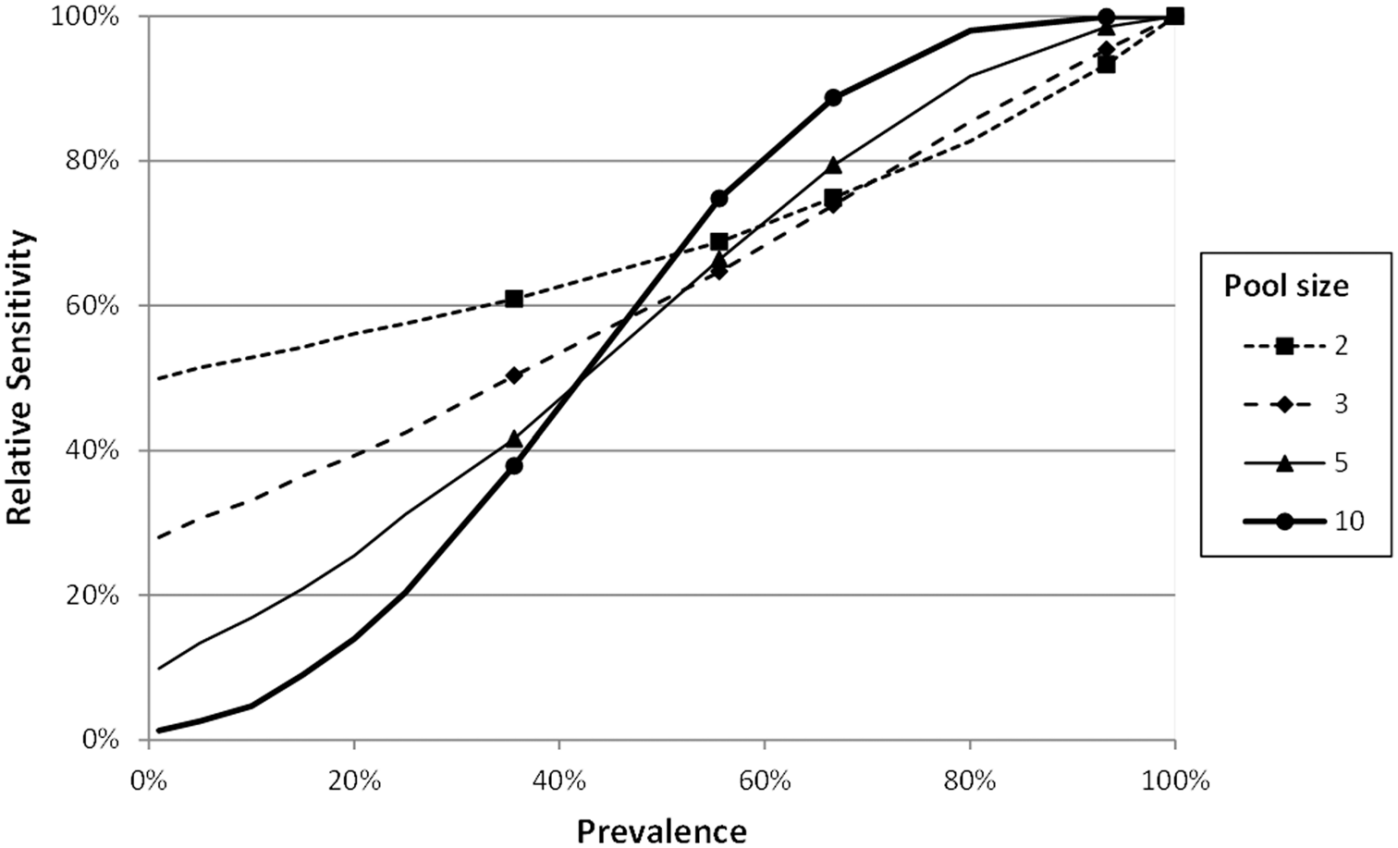
Relative sensitivities corresponding to a pooled diagnostic for PRRSv using low load samples of *Ct* (*mCt* = 36.0, *sCt* = 1) (data from Gerber et al, 2013)[8].

However, these authors described a group of samples with low pathogen load (*mCt* = 36, *sCt* = 1). In the acute phase the relative sensitivity was greater than 90% but the marks corresponding to the 1, 14 and 21 days p.i. (early infection and recovery) where it was from 40 to 80% (Figure 4). And also this is consistent with the results of [8].

The use of pooled samples could be a good strategy in order to reduce analytical cost in surveillance programmes, but loss of sensitivity could be a critical issue due to existence of false negative results.

By way of conclusion, the effect of 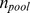 on the relative sensitivity depends on such as the values of the prevalence as the quantity of pathogen load.
